# Neuronal and glial markers are differently associated with computed tomography findings and outcome in patients with severe traumatic brain injury: a case control study

**DOI:** 10.1186/cc10286

**Published:** 2011-06-24

**Authors:** Stefania Mondello, Linda Papa, Andras Buki, M Ross Bullock, Endre Czeiter, Frank C Tortella, Kevin K Wang, Ronald L Hayes

**Affiliations:** 1 Department of Anesthesiology, University of Florida, 1600 S.W. Archer Road, Gainesville, FL 32610-0254, USA; 2 Clinical Department, Center of Innovative Research, Banyan Biomarkers, Inc., 13400 Progress Blvd, Alachua, FL 32615, USA; 3 Department of Emergency Medicine, Orlando Regional Medical Center, 86 W. Underwood Street, S-200, Orlando, FL 32806, USA; 4 Department of Neurosurgery University of Pecs, 2 Rét street, H- 7624 Pecs, Hungary; 5 Department of Neurosurgery University of Miami, 1095 NW 14th Ter, Miami, FL 33136-1060, USA; 6 Department of Applied Neurobiology, Division of Psychiatry and Neuroscience, Walter Reed Army Institute of Research, 503 Robert Grant Ave, Silver Spring, MD 20910-7500, USA; 7 Diagnostic Research and Development Department, Center of Innovative Research, Banyan Biomarkers, Inc., 12085 Research Drive, Alachua, FL 32615, USA; 8 Center for Neuroproteomics and Biomarkers Research, Department of Psychiatry, McKnight Brain Institute, University of Florida, 100 S. Newell Drive Bldg. 59, Gainesville, FL 32611, USA; 9 Department of Clinical Programs, Center of Innovative Research, Banyan Biomarkers Inc., 13400 Progress Blvd, Alachua, FL 32615, USA

**Keywords:** Traumatic Brain Injury, Biomarkers, Diagnostic, Outcome, Computed tomography

## Abstract

**Introduction:**

Authors of several studies have studied biomarkers and computed tomography (CT) findings in the acute phase after severe traumatic brain injury (TBI). However, the correlation between structural damage as assessed by neuroimaging and biomarkers has not been elucidated. The aim of this study was to investigate the relationships among neuronal (Ubiquitin carboxy-terminal hydrolase L1 [UCH-L1]) and glial (glial fibrillary acidic protein [GFAP]) biomarker levels in serum, neuroradiological findings and outcomes after severe TBI.

**Methods:**

The study recruited patients from four neurotrauma centers. Serum samples for UCH-L1 and GFAP were obtained at the time of hospital admission and every 6 hours thereafter. CT scans of the brain were obtained within 24hrs of injury. Outcome was assessed by Glasgow Outcome Scale (GOS) at discharge and at 6 months.

**Results:**

81 severe TBI patients and 167 controls were enrolled. The mean serum levels of UCH-L1 and GFAP were higher (p < 0.001) in TBI patients compared to controls. UCH-L1 and GFAP serum levels correlated significantly with Glasgow Coma Scale (GCS) and CT findings. GFAP levels were higher in patients with mass lesions than in those with diffuse injury (2.95 ± 0.48 ng/ml versus 0.74 ± 0.11 ng/ml) while UCH-L1 levels were higher in patients with diffuse injury (1.55 ± 0.18 ng/ml versus 1.21 ± 0.15 ng/ml, p = 0.0031 and 0.0103, respectively). A multivariate logistic regression showed that UCH-L1 was the only independent predictor of death at discharge [adjusted odds ratios 2.95; 95% confidence interval, 1.46-5.97], but both UCH-L1 and GFAP levels strongly predicted death 6 months post-injury.

**Conclusions:**

Relationships between structural changes detected by neuroimaging and biomarkers indicate each biomarker may reflect a different injury pathway. These results suggest that protein biomarkers could provide better characterization of subjects at risk for specific types of cellular damage than that obtained with neuroimaging alone, as well as provide valuable information about injury severity and outcome after severe TBI.

## Introduction

Accurate determination of the initial severity of primary brain damage after severe head injury is crucial for establishing neurologic prognosis and to balance the risks and benefits of treatment [1]. Outcome prediction remains difficult because neurologic assessment is often influenced by the use of sedatives, analgesics, or muscle relaxants. Assessment of structural damage by neuroimaging is not influenced by these confounders. Marshall and colleagues [2] proposed a descriptive system of computed tomography (CT) classification, which focuses on the presence or absence of a mass lesion and differentiates diffuse injuries by signs of increased intracranial pressure. However, the Marshall classification has limitations. This classification system might mask patients who have diffuse axonal injury (DAI) or signs of raised intracranial pressure (ICP) in addition to a mass lesion, and does not fully use the prognostic information contained in the individual CT characteristics scored [3]. Furthermore, CT has not reliably predicted outcome and can only capture momentary snapshots of the dynamically evolving process of TBI, and important lesions that occur at the microscopic level cannot be visualized.

Biologic markers that reliably reflect either the extent of brain damage and microscopic pathological events and are easy to measure (i.e., in peripheral blood) have long been sought [4]. Biomarkers might mirror evolving processes in the brain that occur at the microscopic level as well as track pathophysiological mechanisms that underlie damage following severe traumatic brain injury (TBI).

Ubiquitin C-terminal Hydrolase-L1 protein (UCH-L1), highly enriched and specifically expressed in neurons, is involved in either the addition or removal of ubiquitin from abnormal proteins including misfolded proteins, and proteins damaged by oxidation or denatured by other means, that are destined for proteasomal degradation [5]. A recent study reported that levels of UCH-L1 in cerebrospinal fluid (CSF) were significantly increased in severe TBI and found a significant association with severity measures [6].

Glial fibrillary acidic protein (GFAP) is a monomeric intermediate filament protein of astrocytes and is considered specific for central nervous system (CNS) disease. Recently, other reports have confirmed that serum GFAP is a specific marker of brain damage after head trauma [7,8].

The objectives of our study were: to evaluate early values and trends of biomarker levels and associations with neuroradiological findings assessed by the Marshall score; to determine the prognostic value of serum concentrations of UCH-L1 and GFAP for clinical outcome at discharge and at six months; and to assess if the association of different biomarkers and clinical indices of severity could be a more a powerful predictor of outcome after severe TBI than clinical indices alone.

## Materials and methods

### Study sites, design, and population

Approval of this study was obtained from the local ethics committee of all the sites involved and from the Western Institutional Review Board and Human Research Protection Office. Eighty-one adult patients presenting to the University of Pécs (*n *= 30), University of Szeged (*n * = 21), and University of California Davis (*n * = 20), and from the University of Maryland Shock Trauma Center (*n * = 10) with severe head injury were included in this prospective multicenter study. Sixty-one patients presented with isolated head injury. Twenty severe TBI patients had minor concomitant injury of the thorax and⁄or abdomen and/or extremities. Multiple trauma were excluded (see exclusion criteria, below). Written informed consent was obtained from a next of kin because all eligible patients were in coma within 24 hours from the admission. Exclusion criteria were no informed consent, patients younger than 18 years of age, female patients that were or may have been pregnant, known history of neurological disease, and Injury Severity Score (ISS) greater than 15. Inclusion criteria were a Glasgow Coma Score (GCS) score of eight or less on presentation. Treatment of patients, according to international guidelines, was targeted at a normal ICP and maintaining cerebral perfusion pressure [9]. Initial CT scans obtained on admission were analyzed according to the classification of Marshall and colleagues [2]. CT scans were interpreted by a qualified neuroradiologist at a central location. For the purpose of our analysis, Marshall was further classified into two groups using dichotomized categories (diffuse injury versus focal mass lesion), as previously described [10]. Outcome was assessed as in-hospital mortality and at six months using the Glasgow Outcome Scale (GOS) [11].

In addition, 167 healthy blood donors were enrolled, during a one-year period, to establish normal serum UCH-L1 and GFAP levels.

### Analysis of UCH-L1 and GFAP

Venous blood samples were taken at hospital admission (median seven hours) and every six hours thereafter. For the purpose of the analysis and to handle potential missing samples, time after injury for each sample was calculated and the samples were separated into 12 or 24-hour time period. Approximately 5 mL of serum was collected from each subject at each sample point. Samples were centrifuged for 10 minutes at 4,000 rpm and immediately frozen and stored at -70°C until the time of analysis. Samples were measured using a standard UCH-L1 sandwich ELISA protocol as described below. Reaction wells were coated with capture antibody (500 ng/well purified anti-rabbit UCHL1, made in-house) in 0.1 M sodium bicarbonate, pH 9 and incubated overnight at 4°C. Plates were then emptied out and 300 μl/well blocking buffer (Startingblock T20-TBS) was added and incubated for 30 minutes at ambient temperature with gentle shaking. This was followed by addition of antigen standard (UCHL1 standard curve: 0.05 to 50 ng/well) unknown samples (3 to 10 uL CSF) or assay internal control samples. The plate was incubated for two hours at room temperature then washed using an automatic plate washer (each well rinsed with 5 × 300 μl with wash buffer (TBST)). Detection antibody (anti-rabbit UCH-L1-HRP conjugation, made in-house at 50 μg/mL) in blocking buffer was then added to wells at 100 μl/well and the plates were further incubated for 1.5 hours at room temperature. After additional automatic washing, biotinyl-tyramide solution (Elast ELISA Amplification Kit, PerkinElmer, Inc., Waltham, MA, USA) was added and the plate was incubated for 15 minutes at room temperature followed by automatic washing. Addition of streptavidin-HRP (1:500, 100 ul/well) in PBS with 0.02% Tween 20 and 1% BSA for 30 minutes incubation at room temperature was followed by automatic washing. Finally, the wells were developed with substrate solution: Ultra-TMB ELISA 100 ul/well (Pierce# 34028) with incubation for 5 to 30 minutes and read at 652 nm with a 96- well spectrophotometer (Spectramax 190, Molecular Device, Sunnyvale, CA, USA). GFAP protein was analyzed using a commercially available (Biovendor Laboratory Medicine Inc., catalog n° rd192072200, Brno, Czech Republic) polyclonal two-side immunoluminometric assay according to the manufacturer's instructions. A standard curve was constructed by plotting absorbance values versus GFAP concentrations of calibrators.

### Statistical methods

The Mann-Whitney test was used to test differences between groups in glial and neural proteins, separately for significance in case of two groups and the Kruskal-Wallis test was used in case of three or more groups. Spearman correlations were used to test correlations between clinical variables and biomarkers. Univariate logistic regression was used to evaluate the prognostic ability of the clinical and biochemical variables, separately, to predict the probability of being deceased (GOS = 1) at discharge and six months after severe TBI. c-statistic (the area under an receiver operating characteristic (ROC) curves) provides an overall measure of classification accuracy (representing the overall proportion of individuals correctly classified), with the value of 1.0 representing perfect accuracy. A ROC curve was created to explore the ability of the biomarker to construct reasonable cutoff values for these variables to predict survival. The optimal cutoff value was chosen as the best operating point. Crude odds ratios (OR) with 95% confidence intervals (CIs) are presented. The Marshall classification was treated as an ordered variable, and the GCS was treated as continuous variables. Multivariate logistic regression with forward stepwise selection procedures was used to identify variables that contributed independently to the risk of being deceased at discharge or six months after severe TBI. Adjusted ORs with 95% CIs are presented. All tests were two-tailed. A *P *value less than 0.05 was considered significant. The software package SAS 9.2 (SAS Institute, Inc., Cary, NC, USA) was used for the statistical analyses.

## Results

The clinical and demographic characteristics of the 81 patients included in this study are summarized in Table 1. Mean UCH-L1 serum concentrations in the first 24 hours in the TBI patients were 1.36 ± 0.11 ng/ml and 0.12 ± 0.02 ng/ml in controls (*P *< 0.0001), and for GFAP it was 2.01 ± 0.29 ng/ml versus 0.07 ± 0.03 ng/ml (*P *< 0.0001). UCH-L1 concentrations were strongly correlated with GFAP (R = 0.53, *P *< 0.001).

**Table 1:** Summary of demographic and clinical characteristics

		Severe TBI (*n * = 81)	Controls (*n * = 167)
**Age, y, mean (SD)**		47.89 ± 20.35	36.91 ± 14.06
**Gender, n (%)**	Female	16 (19.75)	72 (43.1)
	Male	65 (80.25)	95 (56.9)
**Race, n (%)**	Asian	3 (3.7)	6 (3.6)
	Black or African American	7 (8.64)	26 (15.6)
	Caucasian	70 (86.42)	134 (80.2)
	Other	1 (1.23)	1 (0.6)
**Ethnic, n (%)**	Hispanic or Latino	2 (2.47)	13 (7.8)
	Not Hispanic or Latino	79 (97.53)	146 (87.4)
	Other	-	8 (4.8)
**Injury Mechanism, n (%)**	Motor Vehicle Accident	30 (37.04)	-
	Motor Cycle Accident	3 (3.7)	-
	Gun Shot Wound	2 (2.47)	-
	Fall	32 (39.51)	-
	Assault	4 (4.94)	-
	Other	10 (12.35)	-
GCS, median (range)*		5 (3-8)	15
**Marshall Score, n (%)**	Diffuse Injury I	1 (1.23)	-
	Diffuse Injury II	20 (24.69)	-
	Diffuse Injury III	9 (11.11)	-
	Diffuse Injury IV	6 (7.41)	-
	Evacuated Focal Mass Lesion V	4 (4.94)	-
	Focal Mass Lesion VI	41 (50.62)	-
**Outcome**			-
**In hospital, n (%)**	Deceased (GOS 1)	28 (34.57)	-
	Alive (GOS 2-5)	53 (65.43)	-
**6 months, n (%)**	Deceased (GOS 1)	35 (45.44)	-
	Alive (GOS 2-5)	42 (54.56)	-
	Missing	4	-

* At the time of admission

GCS: Glasgow Coma Scale score; GOS: Glasgow Outcome Scale; TBI: traumatic brain injury.

Furthermore, the mean serum UCH-L1 and GFAP concentrations were higher in patients who died compared with patients who were alive at six months post-injury (UCH-L1 1.6 ± 0.22 ng/ml versus 0.65 ± 0.07 ng/ml, *P *= 0.01, and GFAP 3.3 ± 0.57 ng/ml versus 0.36 ± 0.14 ng/ml *P *< 0.001, Mann-Whitney U test).

Serum UCH-L1 and GFAP levels obtained within 12 hours post- injury were higher in subjects with a GCS 3 to 5 (*n * = 47) than in those with a GCS 6 to 8 (*n * = 34) as recorded on the admission (*P *= 0.01 and *P *= 0.001, respectively, test based on the Mann-Whitney statistic).

Thirty-six patients (44%) presented with diffuse injury (Marshall I to IV) and 45 patients (56%) with focal mass lesions (Marshall V to VI) on initial CT scan (Table 1). The average values of UCH-L1 in the first 24 hours post-injury were 1.55 ± 0.18 ng/ml in diffuse injury and significantly lower (1.21 ± 0.15 ng/ml) in focal mass lesion (*P *= 0.01). In contrast, GFAP levels were significantly higher in patients with focal mass lesion (Marshall V to VI) compared to patients with diffuse injury (Marshall I to IV) (2.95 ± 0.48 ng/ml versus 0.74 ± 0.11 ng/ml, *P *= 0.003, test based on Mann Whitney U).

**Figure 1:**
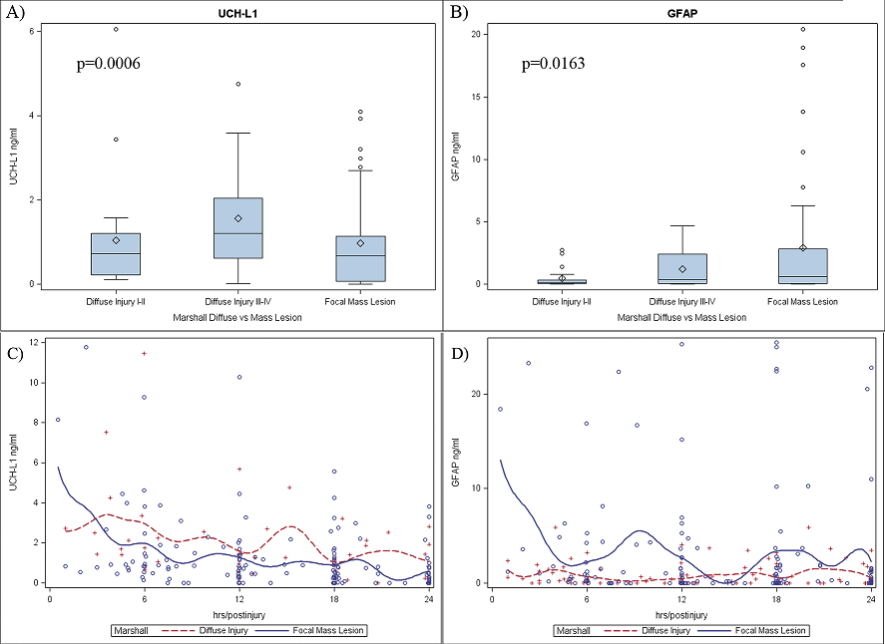
**Serum levels of ubiquitin C-terminal hydrolase-L1 protein (UCH-L1) and glial fibrillary acidic protein (GFAP) in patients with diffuse injury or focal mass lesion**. **(a and b) **Box plots of biomarker boxes enclose 25th to 75th percentiles; vertical whiskers extend to 10th and 90th percentiles. Median (50th percentile) is marked by the line inside the box; mean is marked by the diamond; outliers are plotted with circles. (**a) **Serum levels of UCH-L1 versus Marshall Score (Diffuse Injury vs Focal Mass Lesion) (Kruskal-Wallis Test, *P *= 0.0006). (**b) **Serum levels GFAP versus Marshall Score (Diffuse Injury vs Focal Mass Lesion) (Kruskal-Wallis Test, *P *= 0.0163). **(c and d) **Scatterplot with average smoothing superimposed on a graph of **(c) **UCH-L1 and in **(d) **GFAP levels versus time. The figure was generated using the LOWESS (LOcally WEighted Scatterplot Smoother) technique to draw a smooth line representing the average value of the variable on the y-axis as a function of the variable on the x-axis.

**Figure 2:**
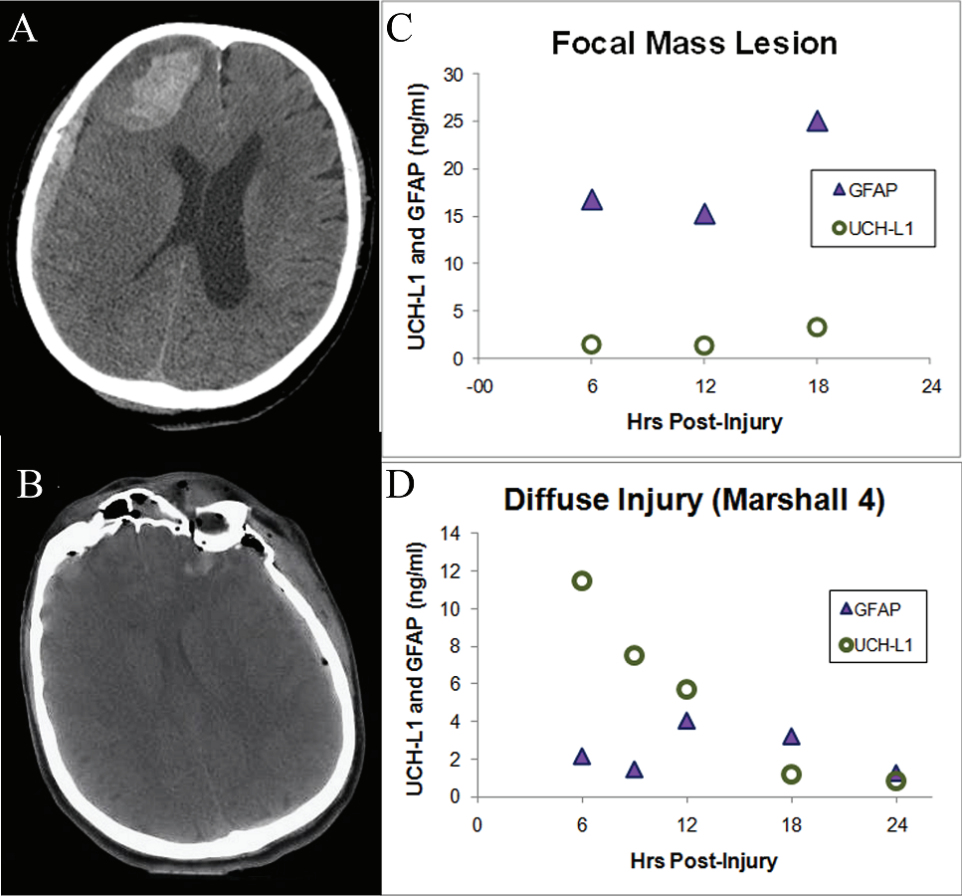
**Ubiquitin C-terminal hydrolase-L1 protein (UCH-L1) and glial fibrillary acidic protein (GFAP) dynamics in human serum after acute injury**. **(a and b) **Computed tomography scans demonstrating focal and diffuse brain injury in individual patients. **(c and d) **UCH-L1 (green circles) and GFAP (blue triangles) serum concentrations measured every six hours in corresponding individual patients. Time (x axis) reflects interval after injury.

Marshall Classification was also categorized into three groups. Diffuse injury I to II, diffuse injury III to IV, and focal mass lesion. The average levels of UCH-L1 in the first 24 hours post-injury were 1.27 ± 0.23 ng/ml, 1.93 ± 0.3 ng/ml and 1.22 ± 0.15 ng/ml. Average GFAP levels were 0.56 ± 0.12 ng/ml, 1 ± 0.2 ng/ml and 2.95 ± 0.48 ng/ml (*P *= 0.0006 and *P *= 0.016, respectively, Kruskal-Wallis test) (Figures 1a and 1b). Two main different patterns were recognized in diffuse injury versus focal mass lesions for both UCH-L1 and GFAP levels in the first 24 hours post-injury (Figures 1c and 1d). GFAP levels were higher in patients with mass lesion than in patients with diffuse injury (*P *= 0.006), whereas UCH-L1 levels were significantly higher in patients with diffuse injury than in patients with mass lesion (*P *= 0.01, Mann Whitney U test). An example of injury patterns in individual patients with diffuse injury or a mass lesion is provided in Figure 2.

Levels of UCH-L1, but not levels of GFAP were weakly correlated with age in patients with severe TBI (correlation coefficient = -0.22, *P *= 0.04).

**Figure 3:**
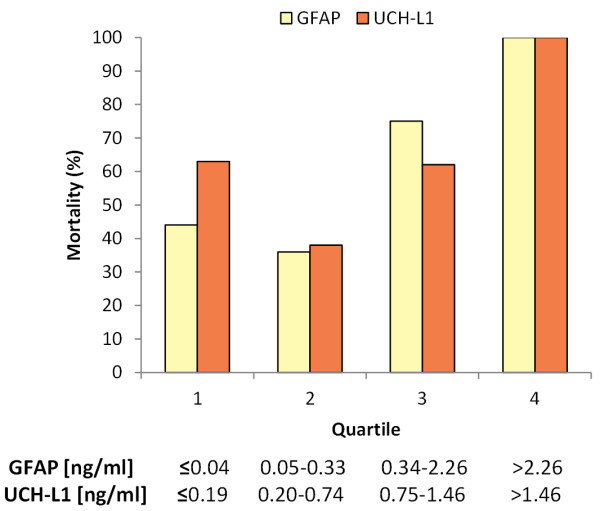
**Serum levels of ubiquitin C-terminal hydrolase-L1 protein (UCH-L1) and glial fibrillary acidic protein (GFAP) in relation to 6 months mortality after severe traumatic brain injury**. Mortality increased with increasing (UCH-L1) and (GFAP) levels (average serum levels in the first 24 hours).

At six month follow up, the mortality rate was 45.44%. Table 2 shows the crude ORs with the 95% CIs of serum biomarkers and GCS, CT characteristics classified by Marshall score, age, and gender for the prediction of death (GOS 1): either in hospital mortality or at six months. The c-statistic (representing the overall proportion of individuals correctly classified) was higher for the biomarkers compared with the other variables for survival prediction at discharge. Figure 3 shows that patients with high biomarker levels in the first 24 hours had an increased mortality at six month after injury.

**Table 2:** Crude OR of clinical and biochemical variables for death (GOS 1) in hospital and six months after severe traumatic brain injury using univariate logistic regression

	*In Hospital *Mortality		6 months Mortality	
**Variable**	**OR (95%CI)**	**C**	**OR (95%CI)**	**C**
**UCH-L1**	2.74 (1.54-4.90)*	0.74	2.29 (1.07-4.90)†	0.62
**GFAP**	1.41 (1.08-1.84)†	0.76	3.30 (1.21-8.50)†	0.75
**Age**	1.02 (0.99-1.05)	0.60	1.07 (1.03-2.11)*	0.78
**GCS**	0.64 (0.45-0.91)†	0.69	0.59 (0.41-0.85)†	0.72
**Gender**				
Female	Reference		Reference	
Male	1.01 (0.80-1.29)	0.60	0.69 (0.19-2.58)	0.53
**Marshall Score**				
I-II	Reference		Reference	
III-IV	9.80 (1.50-63.83)†		12 (1.58-91.08)†	
V-VI	2.80 (0.55-14.23)	0.67	5.56 (1.25-24.77)†	0.67

C = The area under an receiver operating characteristic curve (also known as c-statistic) provides an overall measure of classification accuracy (representing the overall proportion of individuals correctly classified), with the value of one representing perfect accuracy.

* *P *< 0.001, † *P *< 0.01

GCS: Glasgow Coma Scale score; GFAP: glial fibrillary acidic protein; GOS: Glasgow Outcome Scale; OR: odds ratios; UCH-L1: Ubiquitin C-terminal Hydrolase-L1 protein.

**Figure 4:**
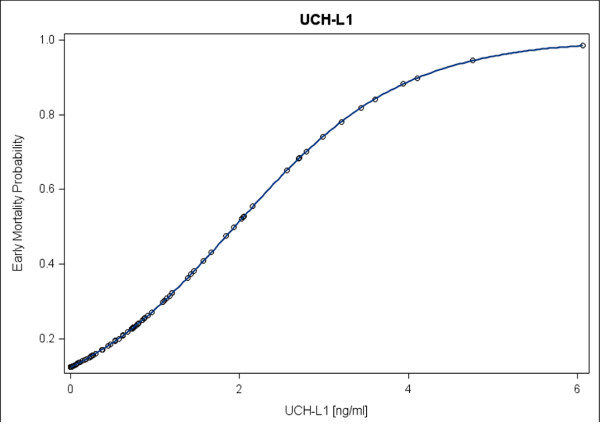
**Spline function analysis showing the association between the serum ubiquitin C-terminal hydrolase-L1 protein (UCH-L1) levels and in hospital mortality, probability of mortality**. The risk of mortality increases slowly up to a "threshold" range of UCH-L1, followed by a more rapid increase and a subsequent levelling off.

For the outcome of in-hospital mortality, multivariate logistic regression analysis revealed that from all variables used in the selection procedure UCH-L1 was the only independent predictor (*P *< 0.05). This result may not be surprising because we found highly significant correlations between UCH-L1 and GFAP. UCH-L1 also significantly associated with GCS and Marshall score. In addition, spline function analysis shows the risk of in-hospital mortality correlated to UCH-L1 serum levels (Figure 4).

As we were specifically interested in discriminating in-hospital mortality optimal cutoff values were calculated by using the criteria of equal-cost-of-misclassification. The specificity in predicting in-hospital mortality of both biomarkers was very high although the sensitivity was low (Table 3).

**Table 3:** Predictive value of serum UCH-L1 and GFAP levels for death (GOS 1) in hospital after severe traumatic brain injury

		*In Hospital *Mortality			
**Variable**	**Cutoff**	**Sensitivity**	**Specificity**	**PPV**	**NPV**
**UCH-L1**	1.89	0.52	0.96	0.52	0.72
**GFAP**	1.44	0.67	0.86	0.72	0.82

GFAP: glial fibrillary acidic protein; GOS: Glasgow Outcome Scale; NPV: negative predictive value; PPV: positive predictive value; UCH-L1: ubiquitin C-terminal hydrolase-L1 protein.

For six months mortality, a multivariate logistic regression analysis revealed that age was the most significant independent predictor (OR 1.12, 95% CI 1.05 to 1.19, *P *= 0.001) followed by UCH-L1 (OR 8.21, 95% CI 1.73 to 38.92, *P *= 0.01) and the GCS (OR 0.58, 95% CI 0.35 to 0.95, *P *= 0.04, c = 0.94). GFAP did not have an additional contribution when UCH-L1 was already entered in the model. For this outcome, GFAP was found have a similar predictive value to UCH-L1 (c = 0.93), where age was the most significant independent predictor (OR 1.1, 95% CI 1.08 to 1.18, *P *= 0.002) followed by GFAP (OR 4.88, 95% CI 1.37 to 17.36, *P *= 0.01) but GCS dropped out of model (OR 0.64, 95% CI 0.38 to 1.09, *P *= 0.1).

**Figure 5:**
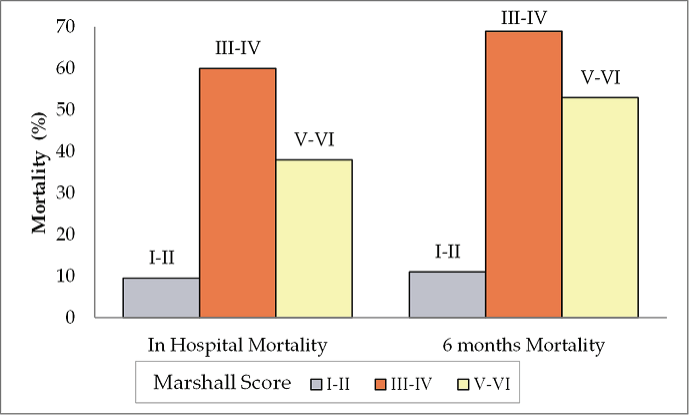
**Associations between Marshall Score and mortality at discharge and six months**. The percentage mortality was low in patients with Marshall Score I and II. The highest mortality rate was observed in patients with Marshall Score III-IV for both in hospital mortality and at six months.

Finally, Figure 5 shows the increased mortality risk in patients with Marshall Score III to IV, consistent with the observation that UCH-L1 (which is elevated more than GFAP in these patients) is an important predictor of mortality.

## Discussion

This prospective study in 81 patients with severe TBI demonstrates that assessment of injury severity and prediction of outcome after severe TBI may be improved with the determination of serum levels of brain specific proteins (UCH-L1 and GFAP) during the acute phase of injury.

The classification of initial CT findings developed by Marshall and colleagues [2] is widely accepted as a classification that allows an evaluation of injury severity in the acute setting. In agreement with previous studies, we found a correlation between the initial serum UCH-L1 and GFAP level and the intracranial diagnosis assessed by Marshall score [6,8].

Importantly, this is the first study to report significantly different pathways for UCH-L1 and GFAP to different type of brain injured patients as characterized by neuroimaging (diffuse injury versus focal mass lesion) (Figures 1c and 1d), including the finding that the two biomarkers had different temporal profile in the same type of injury.

GFAP serum concentrations were higher in patients with with focal mass lesion (Marshall V to VI) than in patients with diffuse injury (Marshall I to IV). In focal mass lesion, GFAP levels tended to remain abnormally elevated over time. In diffuse injury, GFAP levels were relatively low tended to remain low over the monitoring period (Figure 1d). Conversely, in patients with mass lesion UCH-L1 levels were initially higher than in patients with diffuse injury and tended to normalize over the monitoring period. In diffuse injury, UCH-L1 levels were initially high but tended to increase over time or remain abnormally elevated (Figure 1c).

Different patterns of biomarker release imply that different patterns of structural damage involve different pathophysiological mechanisms and may require different therapeutic approaches. Biomarkers analyzed in our study were both brain-specific proteins, UCH-L1 is a neuronal protein while GFAP is a glial protein. Mass lesions seem to result in more damage to glia (physical support for neurons), whereas diffuse injury shows more neuronal injury (functional cells in the brain). It is, in fact, widely accepted that the neurons are highly sensitive to increased ICP or decreased cerebral perfusion pressure inflicted by both primary trauma and secondary insults [12-15]. The assessment of the pathobiological significance of different biomarker pathways and their clinical relevance in patients with TBI has to be addressed in future animal and human studies.

Patients in this study showed imaging changes consistent with morphopathological patterns of diffuse and focal neuronal injury identified following human and experimental TBI [12,13,16]. Changes in biomarker levels can provide real-time complementary information on pathophysiological events that occur in living brain following a brain injury. Moreover, as brain damage is a common finding in cases of mild and moderate head trauma with a broad spectrum of outcomes ranging from mild post-concussive syndrome [17,18] to moderate and severe disability [19], a better understanding of specific profiles of cellular damage could be critical aiding in clinical decision making and lead to more effectively targeted therapy.

Interestingly, we found greater increases in levels and frequency of secondary elevations or sustained high levels within the first 24 hours of both GFAP and UCH-L1 in the focal mass lesion group than diffuse injury (tracked by both biomarkers), suggesting biomarker levels can be associated with adverse physiological events following TBI. Although, the presence of secondary increases in biomarker levels has been often related to secondary insults (hypoxia, hypotension, hyperthermia) [20], in this group an alternative hypothesis is that secondary increases in a specific time (12 to 18 hours) might also be related to brain surgery.

We also analyzed the relation of UCH-L1 and GFAP to outcome and compared the accuracy of UCH-L1 and GFAP for prediction of mortality after TBI.

UCH-L1 was identified as the only independent predictor of early mortality in a model where GFAP, patient characteristics (age, sex) and TBI characteristics (CT findings, GCS) were included as covariates. We believe that this finding is the consequence of the collinearity between UCH-L1, GFAP, and the clinical characteristics. Using a cutoff value of 1.89 ng/ml there was a high specificity of 96% and a sensitivity of 52% for predicting in-hospital mortality. The low sensitivity may be explained by several reasons. In this study, only UCH-L1 levels within 24 hours post-injury were taken for analysis. Thus, unfavorable outcomes due to later secondary brain insults and other complications potentially contributing to poor outcome could not be detected by UCH-L1. However, the high specificity indicates that initial serum UCH-L1 may have a potential value as a marker for major initial brain damage severity.

In agreement with previous studies [21,22], we found a strong prognostic effect of age on mortality at six months (OR 1.07, 95% CI 1.03 to 2.11). Interestingly, although age was the strongest predictor of six months mortality, it was not related to mortality prior to discharge. A multivariate analysis demonstrated that UCH-L1 together with age and GCS results in the best predictive model, with a classification accuracy of 94% in dead versus alive after six months. After age, UCH-L1 was the strongest predictor of mortality at six months. Moreover, we found that GFAP with age may also be an important predictor of outcome at six months after injury when UCH-L1 was not included in the model. This result is consistent with previous studies that have described before the predictive value of GFAP [23]. As reported by Wardlaw and colleagues [24], we found that the Marshall CT classification did not remain a significant independent outcome predictor on multivariate analysis when clinical features and (in our study) biomarkers were included.

UCH-L1 was the only independent predictor of early mortality and, after age, was the strongest predictor of mortality at six months. In contrast to the robust predictive value of UCH-L1, GFAP, and UCH-L1 showed very different profiles in patients having mass lesions compared with those having diffuse injuries. However, these data are consistent with our observation (Figure 5) and the report of Maas and colleagues [3] that diffuse brain injury patients (having relatively higher values of UCH-L1 vs GFAP in our study) had higher mortality rates at discharge and six months than patients with mass lesion (Marshall score V to VI).

It is also important to consider why diffuse brain injury or mass lesions differently affect neurons compared with astroglia. The difference is related to two important distinctions between neurons and glia. First, astrocytes are the most numerous cell type in the central nervous system, with an astrocyte-to-neuron ratio that can reach 10:1 in most brain regions [25]. Second, astrocytes are generally more resistant to ischemia and other stressors than are neurons and unlike neurons, are not vulnerable to glutamate excitotoxicity [26]. In diffuse injury, as a consequence of acceleration/deceleration forces, we postulate a predominance of neuronal cell death resulting in higher levels of UCH-L1 release. In contrast focal mass lesions are characterized by pan-necrosis of glial and neuronal elements, and a relative predominance of glial cell death with higher levels of GFAP. In addition, a reactive astrogliosis in order to isolate the lesion area can occur within a few hours of brain injury. This evolutionarily conserved function might also contribute to increased GFAP levels [26].

Our study shows a significant correlation between serum brain specific protein levels and the hospital admission GCS. When the correlation of either UCH-L1 and GFAP with the GCS is considered, a positive correlation was found by some authors [6,27] which could not be confirmed by others [28,29].

UCH-L1 correlated with age in severe TBI patients. We also examined the correlation among UCH-L1 and GFAP levels and age in our control group that was not found statistically significant.

In our study, 41 (51%) of severe TBI patients presented with a non-evacuated mass lesion (Marshall score VI) at the first CT. The high rate may be explained by the time interval between the injury and the neuroimaging assessment. Indeed 28 of these patients had a second CT scan within the first 24 hours showing an evacuated mass lesion (Marshall score V).

This study has several limitations. It would be interesting to compare the prognostic value of UCH-L1 and GFAP levels and CT findings to measurements of ICP. As ICP measurements were not available in all of our patients, we could not perform such an analysis. Other studies [30,31] have shown that the worst CT scan obtained during the clinical course has greater predictive value. However, the intent of our studies was to investigate the prognostic risk of TBI patients on admission. The predictive analysis examined only in-hospital and six months mortality. For our study, we chose mortality rather than the GOS dichotomized into unfavorable versus favorable since mortality constitutes an objective endpoint. The high mortality (45.44%) observed in our study presumably reflects patients having been more severely injured; a similar rate (49%) was previously reported in the full International Data Bank.

## Conclusions

We report that blood levels of UCH-L1 and GFAP are an indicator of the severity of brain damage after severe head trauma and are correlated with prognosis after trauma. Biomarker profile may be a valuable addition to traditional approach [32] to the TBI patient. The importance of a specific biomarker is related to the clinical question we ask. Moreover, a multimarker strategy might be also useful in refining risk stratification including mortality and morbidity prediction. Finally, different profile of biomarker release after TBI might provide useful tools for meaningfully categorizing patients, who historically have been treated as homogenous groups, into cohorts based on specific pathobiological processes.

## Key messages

• Diffuse injury and focal mass lesion are associated with different biomarker profiles after severe TBI.

• UCH-L1 and GFAP may be considered both as efficient diagnostic and severity biomarker of TBI.

• Serum levels of UCH-L1 and GFAP may be a good prognostic factor for mortality in patients with TBI.

• UCH-L1 and GFAP may provide risk stratifications of the patients and a characterization of the specific types of cellular damage mainly involved in primary injury; potentially leading to a more effectively individualized and targeted therapies.

## Abbreviations

BSA: bovine serum albumin; CI: confidence interval; CSF: cerebrospinal fluid; CT: computed tomography; ELISA: enzyme-linked immunosorbent assay; GCS: Glasgow Coma Scale; GFAP: glial fibrillary acidic protein; GOS: Glasgow Outcome Score; ICP: intracranial pressure; OR: odds ratio; PBS: phosphate buffered saline; ROC: receiver operator characteristic; TBI: traumatic brain injury; UCH-L1: Ubiquitin carboxy-terminal hydrolase L1.

## Competing interests

Drs. Mondello, Papa, Buki, Bullock and Czeiter are consultants of and received consulting fees from Banyan Biomarkers, Inc.; Drs. Wang and Hayes own stock, receive royalties and salaries from, and are officers of Banyan Biomarkers Inc., and as such may benefit financially as a result of the outcomes of this research or work reported in this publication. Banyan Biomarkers, Inc. filled patent applications based upon the disclosure of this publication. Dr. Tortella reports no disclosures.

Material has been reviewed by the Walter Reed Army Institute of Research.

There is no objection to its presentation and/or publication. The opinions or assertions contained herein are the private views of the authors, and are not to be construed as official, or as reflecting true views of Department of the Army or Department of Defense.

## Authors' contributions

SM contributed to data analysis, interpretation of the results and drafted the manuscript. LP, RB and FT participated in manuscript preparation. AB and EC participated in data collection and manuscript preparation. KKW and RLH contributed to the design of the study, participated in the laboratory work and manuscript preparation. All authors have read and approved the article for publication.
